# Lung cancer researchers, 2008–2013: their sex and ethnicity

**DOI:** 10.1007/s11192-015-1785-1

**Published:** 2015-11-12

**Authors:** Grant Lewison, Philip Roe, Richard Webber, Richard Sullivan

**Affiliations:** Institute of Cancer Policy, Guy’s Hospital, King’s College London, Great Maze Pond, London, SE1 6RT UK; Evaluametrics Ltd, 157 Verulam Road, Saint Albans, AL3 4DW UK; Department of Geography, King’s College London, Strand, London, WC2R 2LS UK

**Keywords:** Lung cancer, Onomastics, National origins, Sex

## Abstract

This paper describes the process by which almost all authors of papers in the Web of Science (WoS) can be characterised by their sex and ethnicity or national background, based on their names. These are compared with two large databases of surnames and given names to determine to which of some 160 different ethnic groups they are most likely to belong. Since 2008 the authors of WoS papers are tagged with their addresses, and many have their given names if they appear on the paper, so the workforce composition of each country can be determined. Conversely, the current location of members of particular ethnic groups can be found. This will show the extent of a country’s “brain drain”, if any. Key results are shown for one subject area, and *inter alia* it appears that the majority of researchers of Indian origin who are active in lung cancer research are working in the USA. But East Asians (Chinese, Japanese and Koreans) tend to stay in their country of birth.

## Introduction

There is continuing research interest in the sex and ethnic composition of research personnel. A brief survey of the literature in 2013–2014 indicates that there is a widespread concern about the problems faced by female researchers (no fewer than 24 countries were involved in such research, and there were 71 papers in the 2 years, including several exploring the problems in countries outwith North America and Western Europe, e.g., Gonenc et al. 2013; Homma et al. 2013; Bettachy et al. 2013; Isfandyari-Moghaddam and Hasanzadeh 2013; Garg and Kumar 2014). A major survey of the situation in all the countries of the world and all science was published in *Nature* 2 years ago (Larivière et al. 2013); this showed that women scientists were in the minority in almost all countries, and only achieved parity with men, or a majority, in a few small states.

However there is less interest in the situation of ethnic groups or of “foreign researchers”. The situation in the USA has attracted particular attention from commentators. Because of public opinion against immigrants, bills were introduced in Congress in 1995 that would have imposed restrictions on foreign researchers *inter alia* and required their employers to pay a levy to train US workers (Reichhardt 1995). Successful lobbying by universities and other employers defeated them (Reichhardt 1996). Nevertheless, foreign researchers in the USA do not receive equal treatment (Jaeger et al. 2004; Anon 2004; Dalton 2005) which can make their situation difficult. There also appear to be problems for ethnic minority faculty members in the USA (Griffin et al. 2013; Pololi et al. 2013; Campbell et al. 2013; Hassouneh et al. 2014). In fact, critical attention in the USA is focussed almost entirely on under-represented minorities (African-Americans, Hispanics, and in some cases Native Americans), and hardly at all on the problems that may be encountered by researchers of Asian origins, notably Chinese and Indians, who may have to cope with difficult immigration (Teich 2014), integration and living experiences when they move to the USA. In fact, as we shall see, they are hardly “under-represented minorities” but rather over-represented compared with their presence in the population. [A fuller survey of the relevant prior literature was given in Roe et al. (2014).]

The situation in other countries is also discussed sometimes. Thus several European countries have attracted criticism for being unfriendly to foreigners—France (Coles 1990), Spain (Pickin et al. 2001), Germany (Schiermeier and Wegner 2002) and Italy (Breda 2014). The “brain drain” from developing countries to more advanced ones has received much attention. For example, there are papers on the problems of emigration from Africa (Capuano and Marfouk 2013), the counter effect of subsequent remittances home (Ngoma and Ismail 2013) and the ethics of border controls (Oberman 2013; Ferracioli 2015), but the potential obstacles in the way of researchers who might wish to return home are seldom mentioned (Awofeso 2004; Gungor and Tansel 2014). In East Asia, Japanese industry has been making notable efforts since the 1990s to attract researchers from abroad in an attempt to stimulate its competitiveness (Swinbanks 1993; Ebbesen 1995), and the Japanese government is now stimulating top universities to become more international, both in terms of faculty and students (Normile 2015). China has for some time sought to encourage Chinese researchers who have gone abroad to return (Normile 2006; Xin 2009), but it is also seeking foreigners who can make a distinctive contribution (Qiu 2011). So it appears that East Asia may now be making bigger efforts than Europe and the USA to attract foreign researchers. Are these policies or practices working out in reality?

This paper provides a method whereby the researchers in a given scientific subject area can be characterised by their ethnicity or national background and their sex. This is important for science policy, including the monitoring of the changing roles and positions of women in research and the extent to which a country is welcoming to researchers from abroad and helps them to integrate. It builds on the methods described earlier (Roe et al. 2014) but now allows all the authors on multi-national papers to be classified, and is applicable to all the countries represented in the subject area. Conversely it can reveal the location of researchers of any particular ethnicity or national origin. The methods have been applied to the subject area of lung cancer research, and results for this area are given in some detail, but they can equally be applied to any other research area.

The classification of individuals by ethnicity is hardly an exact science as ethnic origin is by no means an objective fact. If it were so, then individuals who were asked to self-identify would give an accurate answer. Often they would be unable to do so because so many people are now the product of unions between parents of different ethnicity or national origin, so they may be half one group and half another, or from even more groups. (GL’s neighbours used to be English and Japanese, but are now Serbian and Korean; their children probably regard themselves as British.) In practice, the Origins databases used for this study provide not a biological measure or a self-identification but a measure of where people’s names originate. This seems to be a reasonably accurate proxy for a combination of religion, language, culture and ethnicity (Webber 2010).

Attention was focussed on 24 leading countries, responsible for the large majority of global lung cancer research output, as shown in Table 1 with their digraph ISO codes. However, some results are also given for other countries, because the database listed all countries contributing to lung cancer research, and researchers with names characteristic of 90 different countries.

**Table 1:** List of 24 leading countries in lung cancer research, 2004–2013, with ISO digraph codes

Countries	ISO	Countries	ISO	Countries	ISO	Countries	ISO
Australia	AU	Denmark	DK	Japan	JP	Sweden	SE
Austria	AT	France	FR	Netherlands	NL	Switzerland	CH
Belgium	BE	Germany	DE	Norway	NO	Taiwan	TW
Brazil	BR	Greece	GR	Poland	PL	Turkey	TR
Canada	CA	India	IN	South Korea	KR	United Kingdom	UK
China (PR of)	CN	Italy	IT	Spain	ES	USA	US

## Methodology

The file of lung cancer papers (articles and reviews) was obtained from the Web of Science (WoS) for the 6 years, 2008–2013, from the intersection of two “filters”. One was for cancer, and was based on journal names and title words. These included the names of many individual cancers, genes known to pre-dispose people to an enhanced (or reduced) risk of cancer, and specialist drugs and other treatments such as radiotherapy. The other was for lung disease, and consisted of a number of specialist respiratory journals, such as *Experimental Lung Research, Jornal Brasileiro de Pneumologia, Lung* and *Respiration*, and two title words *lung* and trachea*. In addition, all the papers in the journals *Lung Cancer* and *Clinical Lung Cancer* were retained, together with papers with *SCLC* or *NSCLC* in their titles. The file contained details of 22,433 papers.

The analysis of the researchers was based on their combination of surnames and given names. The surnames were compared with our listing of 2.6 million family names which is based on records of the majority of the adult population in the following countries: Australia, Brazil, Canada, Denmark, Germany, Ireland, Italy, Netherlands, Norway, South Africa, Spain, Sweden, the UK and the USA as well as surname frequency distributions for Austria, Belgium, France, India and Japan. For some countries in Eastern Europe and the Middle East, the files were supplemented by data on the names of scientists from these countries found in the WoS. We were able to classify names into over 160 different heritages based on a combination of ethnicity, language and religion, but in this study the classification was simplified to include own country and eight main world regional groups:

- own country (OWN);
- other European country (EUR: Albania, Balkan, Belgium, Bosnia, Britain, Bulgaria, Croatia, Cyprus, Czech, Denmark, Estonia, Finland, France, Germany, Greece, Hungary, Iceland, Ireland, Italy, Latvia, Lithuania, Malta, Montenegro, Netherlands, Nordic, Norway, Poland, Portugal, Romania, Russia, Serbia, Slovakia, Slovenia, Spain, Sweden, Switzerland);
- Latin America (LAT: including Brazil, Guyana and Mexico);
- Levant and Mediterranean (LEV: Algeria, Egypt, Israel, Lebanon, Libya, Morocco, Saudi Arabia, Tunisia, Turkey, Ukraine);
- Africa (AFR: Afrikaaner, Angola, Cameroon, Congo, Eritrea, Ethiopia, Ghana, Ivory Coast, Kenya, Malawi, Mauritius, Nigeria, Sierra Leone, Somalia, Sudan, Uganda);
- South Asia (SAS: Bangladesh, Burma, India, Pakistan, Sri Lanka);
- China (CHI);
- other Asia (ASI: Afghanistan, Armenia, Azerbaijan, Cambodia, Georgia, Iran, Iraq, Japan, Korea, Laos, Malaysia, Mongolia, Nepal, Philippines, Singapore, Thailand, Vietnam);
- other non-European and Oceanic (OCE: Australia, Caribbean, Fiji, Indonesia, New Zealand).

The methodology is more fully described in a recent paper by Roe et al. (2014).

Given names often (but not always) connote the sex of the person, and we have compiled a list of some 0.7 million such names, including some misspellings and phonetic misrepresentations. This has recently been complemented with the given names of all doctors on the UK Medical Register—over 328,000 individuals, many of whom were born or educated in other countries. Some given names connote a different sex in different countries—for example, Andrea is female in the UK but male in Italy. A few countries (in the present study, only Poland) have surnames with gender endings and this can also be used to determine the sex of an author.

In Roe et al. (2014), attention was confined to papers from a single country, but we were now able to identify the names of the authors from each of the countries in a multi-national paper because the WoS lists them with their addresses in the following format:

[Scagliotti, Giorgio V.] Univ Torino, Thorac Oncol Unit, Dept Clin & Biol Sci, S Luigi Hosp, I-10043 Turin, Italy; [Germonpre, Paul] Univ Ziekenhuis Antwerpen, Edegem, Belgium; [Planchard, David] CHU Poitiers, Poitiers, France; [Reck, Martin] Krankenhaus Grosshansdorf, Grosshansdorf, Germany; [Lee, Jin Soo] Natl Canc Ctr Korea, Goyang, South Korea; [Biesma, Bonne] Jeroen Bosch Ziekenhuis, Shertogenbosch, Netherlands; [Szczesna, Aleusandra] Mazowieckie Ctr Leczenia Chorob Pluc & Gruzlicy, Otwock, Poland; [Morgan, Bruno] Leicester Royal Infirm, Dept Radiol, Leicester, Leics, England

although not all the authors have given names that would allow their sex to be determined.

A special macro was written to enable the names of all authors from each of the countries to be listed in appropriate columns of a spreadsheet for each paper. These were then each classified by national group and sex, where available, so that the contributions of each of the national groups and sexes could be determined. However, the main analysis was performed on the long list of 84,533 different names, each of which was associated with a country and had its frequency of occurrence listed. For each of the 24 selected countries, and for the rest of the world (RoW), the composition of the lung cancer research workforce and the contributions (sums of the numbers of papers) from researchers whose names came from each ethnic group (or world region, *v.s.*) were determined.

However, we found during our analysis that some East Asian names belonging to researchers working in China, Japan or South Korea, had been mis-classified as European as they were ambiguous, such as Jung, Lee and Park. It was obvious from the given names of these researchers if they were Asians or Europeans. Thus Jung, Andreas working in Germany was clearly German, but Jung, Deuk-Kju working in South Korea was Korean. Likewise, Park, Bernard J. working in the USA was considered to be of European origin, but Park, Byung-Joo in South Korea was taken as Korean. These were manually corrected, and some other adjustments to ethnicity were made.

It also became apparent that some names with different given names or initials actually referred to the same person. Thus there were only two Aaronsons in our list of researchers, one was Neil and the other Stuart A. Both could be classed as male. Another Aaronson, S.A. was clearly the same as Aaronson, Stuart A, and so could be counted as male. In this way we were able to sex quite a lot of researchers whose given names were missing or incomplete.

## Results: the sex of researchers

The data on the national origins and on the sex of the lung cancer researchers in the 24 selected countries, plus the Rest of the World, were obtained from a large file that looked like Table 2. The top person evidently worked both in China and the USA, and the first and ninth names were sexed by comparison with the row(s) below.

**Table 2:** Small excerpt from the file listing the names of all lung cancer researchers

Name	Country	ISO	Count	Ethnic	Sex	Region
Aakre, J.	USA	US	1	NO	M	EUR
Aakre, Jeremiah	China	CN	1	NO	M	EUR
Aakre, Jeremiah A.	USA	US	4	NO	M	EUR
Aamini, Mahnaz	Iran	IR	1	IR	F	ASI
Aapro, M.	Switzerland	CH	1	FI	X	EUR
Aarab-Terrisse, S.	France	FR	1	MA	X	LEV
Aarndal, Steinar	Norway	NO	2	NO	M	EUR
Aaron, Jesse	USA	US	1	UK	M	EUR
Aarons, Y.	Australia	AU	1	ES	F	EUR
Aarons, Yolanda	Australia	AU	1	ES	F	EUR

For the analysis by sex, all 24 countries, plus the RoW, have been included in Table 3. The table shows the percentages of names that could be sexed, and the percentage of such names that were female. The calculation was made both for the number of researchers (this will be an over-estimate, as in Table 2 there are only 7 people, not 10) and for their total contributions.

**Table 3:** Analysis of lung cancer researchers in 24 leading countries by sex

ISO	Total			Males	Females	Unknown	Sexed (%)		*F*/(*M* + *F*) (%)	
	*P*	*C*	*C/P*	*P*	*P*	*P*	*P*	*C*	*P*	*C*
CN	13,500	29,897	2.21	2241	3918	7341	46	42	63.6	63.9
RoW	5226	8475	1.62	1920	1733	1573	70	74	47.4	45.8
PL	842	1643	1.95	396	348	98	88	91	46.8	43.2
IT	4647	9220	1.98	2060	1802	785	83	87	46.7	39.6
BR	721	911	1.26	338	282	101	86	86	45.5	43.9
ES	2300	4376	1.90	983	808	509	78	81	45.1	42.2
KR	3990	10,533	2.64	938	754	2298	42	43	44.6	44.7
TR	1827	2747	1.50	819	648	360	80	83	44.2	39.0
SE	560	1159	2.07	268	205	93	84	86	43.3	39.7
TW	2867	8243	2.88	508	378	1981	31	34	42.7	38.5
Wld	36,480	77,204	2.12	10,471	10,876	15,139	59	56	50.9	48.5
FR	3319	7976	2.40	1346	946	1027	69	80	41.3	38.2
DK	502	965	1.92	257	179	66	87	90	41.1	44.0
UK	2908	4782	1.64	1403	914	591	80	84	39.4	35.1
US	19,962	44,423	2.23	9854	6416	3692	82	84	39.4	34.9
AU	1101	2336	2.12	531	343	227	79	84	39.2	38.6
GR	1247	2194	1.76	620	369	258	79	85	37.3	31.1
CA	1933	4585	2.37	940	551	442	77	79	37.0	37.1
IN	940	1339	1.42	363	212	365	61	62	36.9	34.3
NO	300	923	3.08	172	95	33	89	93	35.6	26.2
NL	1638	3738	2.28	865	462	311	81	86	34.8	31.1
CH	756	1293	1.71	417	212	127	83	87	33.7	29.6
BE	606	1186	1.96	287	143	176	71	72	33.3	28.9
AT	412	851	2.07	242	105	65	84	89	30.3	23.1
DE	3523	6935	1.97	2083	841	599	83	88	28.8	23.9
JP	8900	24,503	2.75	4260	1703	2937	67	68	28.6	22.1

Countries are ranked by percentage of female researchers

*P* number of people, *C* number of contributions (integer count), *F* number of females, *M* number of males, *RoW* rest of the world

The high percentage of females in China is clearly anomalous as fewer than half the names could be sexed—this was also the case for Taiwan and Korea. Among European countries, Canada and the USA, on average just over 80 % of names could be sexed, and the female percentages are more reliable. The Germanic countries, Belgium and the Netherlands are ranked noticeably low on female participation. On the other hand Poland, a former Communist country where females were strongly encouraged to work (Webster 2001), ranked highly, and the 10 other eastern European countries (the new “accession Member States” of the European Union) as a group ranked more highly still, with an actual majority of female researchers (51.5 %) though their collective contribution was only 46.6 %.

The five South American countries (Argentina, Brazil, Chile, Colombia and Venezuela) also scored highly for female participation with nearly 46 % of researchers and 44 % of contributions, slightly higher than the values for Brazil alone. The three Mediterranean Latin countries (Italy, Portugal and Spain) also scored highly, and Portugal had the highest female participation, with over 61 % of female researchers, whose contribution was 58 %.

The correlation of the percentage of females in the above table (for the 11 countries for which a comparison could be made) with that obtained from another study on cancer screening is quite high (*r*^2^ = 0.63). However the proportion of female researchers in lung cancer averaged only 39 % compared to 46 % for cancer screening. Sweden was an exception, with a higher female percentage in lung cancer (43 %) compared with 40 % for cancer screening.

**Fig. 1:**
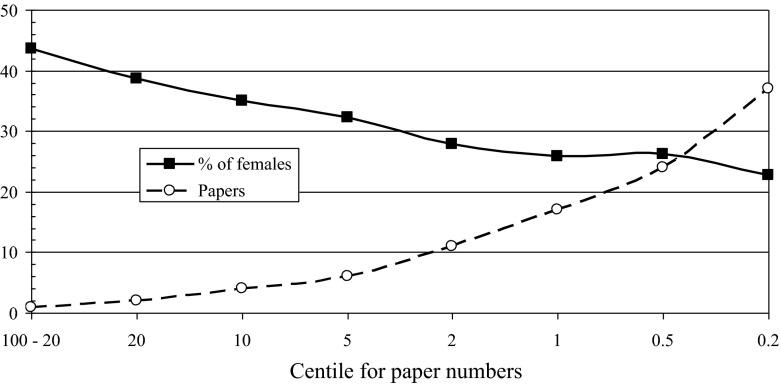
Percentage of female authors whose number of lung cancer papers put them in given centiles of the population of 84,533 authors

The file of lung cancer researchers also enabled us to investigate whether there was a difference between men and women in the numbers of papers that they write. Figure 1 shows the sex ratio *F*/(*M* + *F*) for groups of authors who publish sufficient papers to put them in a given centile. Thus of the 84,533 authors, the top 1 % (*n* = 845) each wrote at least 17 papers, and the figure shows that just under 26 % of those whose sex could be determined were female. By contrast, the 53,143 authors with but a single paper (probably mainly graduate students) were nearly 44 % female. This shows clearly that the percentage of females falls off with output, and it is likely to be strongly correlated with seniority. A similar graph could be produced for individual countries, or ethnic groups, provided that there are enough people in the group or country to make the analysis worth-while.

## Results: the ethnicity or national background of researchers

For the analysis of backgrounds of the researchers, we first determined the percentage of researchers with “own country” ethnicity. Table 4 shows, for each country, the national background(s) of the names that were selected and the corresponding percentages of their numbers and contributions. The result for Brazil is anomalous, as most of its researchers are descended from Europeans and would have European or Latin American names. (A scientific conference in Caxambu of the Brazilian Biochemical Society, which GL attended in 1994, was almost entirely populated by Brazilians who appeared to be of European origin.) If these are allowed as “own country” names, then they would represent 90 % of Brazilian researchers with a contribution of 91 %.

**Table 4:** Numbers and percentages of “own country” lung cancer researchers in 24 leading countries

Country	Own CU	*P* (%)	*C* (%)	Country	Own CU	*P* (%)	*C* (%)
BR	BR	26.4	27.1	NL	NL	62.9	63.8
DK	DK, SC	41.0	41.8	IN	IN	67.8	68.3
CA	FR, UK	42.0	42.9	ES	ES	68.3	67.3
SE	SC, SE	48.2	50.7	DE	DE	70.3	71.2
AU	UK	51.9	55.7	BE	BE, FR, NL	76.2	72.2
NO	NO, SC	55.3	58.8	TW	CN	78.9	74.5
FR	FR, UK	58.5	60.6	PL	PL	80.0	76.7
UK	UK	59.8	60.1	CN	CN	83.7	85.3
US	EUR	60.1	61.4	TR	TR	85.6	86.6
GR	GR	60.5	64.0	IT	IT	90.5	91.2
AT	DE	61.9	59.5	KR	KR	92.4	92.9
CH	DE, FR, IT	62.0	64.9	JP	JP	95.3	96.3

The countries with the greatest percentage of their lung cancer workforce of non-native origin appeared to be the Nordic ones (Denmark, Sweden and Norway), and Canada. The UK also had a high proportion of its lung cancer researchers with non-national ethnic backgrounds (40 %) and the same percentage of contributions. On the other hand, Italy had only 10 % of non-Italians, and Korea and Japan even fewer foreigners (8 and 5 % respectively) though there were rather more in Taiwan (21 %) and in China (16 %). This feature of Italian research was found in a previous study (Roe et al. 2014).

We now consider the contribution of other European researchers to the lung cancer research of the 14 selected European countries. This is shown in Table 5. The results are similar to those of Table 4, except that the UK drops from fifth to tenth place with its proportion of other European nationals among its lung cancer researchers. Its acceptance of non-Europeans is therefore correspondingly greater. There were 7.0 % with a South Asian background, three fifths of them Indian; 4.0 % from other Asian countries, and 3.1 % Chinese. These percentages are much the highest in Europe except that Sweden had a slightly greater percentage of researchers of Chinese origin. The UK also had 2.2 % of lung cancer researchers with North African or Levantine names (third highest in Europe), 0.8 % with African names (second to the Netherlands) and 0.7 % with names from Latin America (highest in Europe). Altogether, its lung cancer research population with non-European names amounted to 19 % of the total.

**Table 5:** Contributions of researchers from other European countries to the lung cancer research of 14 selected European countries

Country	Other EUR (%)		Country	Other EUR (%)		Country	Other EUR (%)	
	*P*	*C*		*P*	*C*		*P*	*C*
DK	52.4	53.8	FR	28.7	29.5	ES	17.3	19.9
NO	36.3	27.1	CH	27.4	25.4	BE	16.7	22.1
SE	35.7	36.1	NL	27.0	27.1	PL	16.4	19.5
GR	33.9	32.2	DE	21.5	21.2	IT	6.6	6.0
AT	33.7	37.4	UK	21.3	21.3			

*P* people, *C* contributions (integer counts)

These percentages can be compared with census data for England and Wales in 2011 (ONS 2012). There were about 5.3 % of “other White” including Irish (corresponding approximately to “other Europeans” in the above table), 2.5 % of Indian origin, 0.7 % of Chinese and 4.2 % of other Asians. So the Chinese were over-represented among lung cancer researchers by 3.1/0.7 = 4.4, the Indians by 4.2/2.5 = 1.7 and other Asians were slightly under-represented by 4.0/4.2 = 0.95. The other Europeans were also over-represented by 21.3/5.3 = 4.0. Many of the Chinese would have been graduate students and would probably have returned to China or gone elsewhere after obtaining their doctorates or other degrees.

Canada and the USA were even more accepting of non-Europeans, and their percentages of the different groups are shown in Table 6. Almost 40 % of US lung cancer researchers were of non-European ethnicity or national background, of whom by far the largest group were Chinese (13.8 % of the US total), followed by Indians (5.8 %) and Koreans (3.5 %). Despite the large numbers of Latin Americans now in the population, they represent only 4.3 % of American lung cancer researchers, even when people with Brazilian, Portuguese and Spanish names are included. US Census data for 2010 show that “Latinos” accounted for well over one third of those living in the USA but born abroad, compared with the Chinese (5 %) and Indians (4 %). However, only 5 % of them had university degrees, compared with 50 % of the Chinese and 74 % of the Indians (US Census Bureau 2012), so it is not surprising that their contribution to lung cancer research was relatively small.

**Table 6:** Percentages of non-European lung cancer researchers in Canada and the USA

	CHI	ASI	SAS	LEV	LAT	AFR	Other	Total
CA	11.0	9.6	5.6	4.2	0.9	0.4	2.7	34.4
US	13.8	9.6	7.7	4.5	1.4	1.0	1.8	39.8

*CHI* Chinese origin, *ASI* other Asian, *SAS* south Asian (Bangladesh, India, Pakistan, Sri Lanka), *LEV* Levant (including north Africa), *LAT* Latin America, *AFR*  rest of Africa

Virtually all Americans, other than some Native Americans, have names that come from other regions of the world so it is useful to use names to identify the parts of the world from which they, or their ancestors, came. Clearly it is impossible for us to distinguish in this way between people born abroad, and second and subsequent generation immigrants. This is not so much a matter of mis-classification as a conceptual problem—when are people better classified as ‘native’ or ‘immigrant’?

The file also allows us to determine where lung cancer researchers with given ethnicities are now based and how much they are contributing to either their countries of origin or their new host countries. We previously found (Basu et al. 2012) that the output of cancer research papers by people of Indian origin now living in Canada and the USA was greater than that of Indians remaining in India. In lung cancer research, of the 2233 researchers with Indian names, over half (1164 or 52 %) are working in the USA and only 637 (28.5 %) in India. There are 124 in the UK, 80 in other European countries, 73 in Canada and 155 elsewhere. The situation is very different for the Chinese, Japanese and Koreans, see Table 7.

**Table 7:** Current locations of lung cancer researchers from China, Japan and South Korea

Ethnicity	Workplace						
	China	Europe	Japan	Korea	USA	Other	Total
CN	11,301	220	124	178	2762	2725	17,310
JP	18	27	8485	9	341	90	8970
KR	1151	40	51	3688	702	443	6075
CN (%)	65.3	1.3	0.7	1.0	16.0	15.7	
JP (%)	0.2	0.3	94.6	0.1	3.8	1.0	
KR (%)	18.9	0.7	0.8	60.7	11.6	7.3	

Clearly, most of these East Asians remain in their own country, although the Chinese travel abroad the most, and the Japanese the least, and hardly at all to China or Korea. There is also very little movement to Japan by Chinese and Koreans, and some of the 51 Koreans working in Japan may be ones whose forebears have been there for several generations. In 2005, there were some 901,000 people of Korean ancestry living in Japan (out of a population of 128 million) or 0.7 %. The percentage of the lung cancer researchers in Japan with Korean names was 0.6 %, which is slightly less. Despite the effort described in the introduction to persuade more non-Japanese to go there to do research, the percentage of Europeans in Japan is still tiny, <2 % (171 out of 8900).

**Fig. 2:**
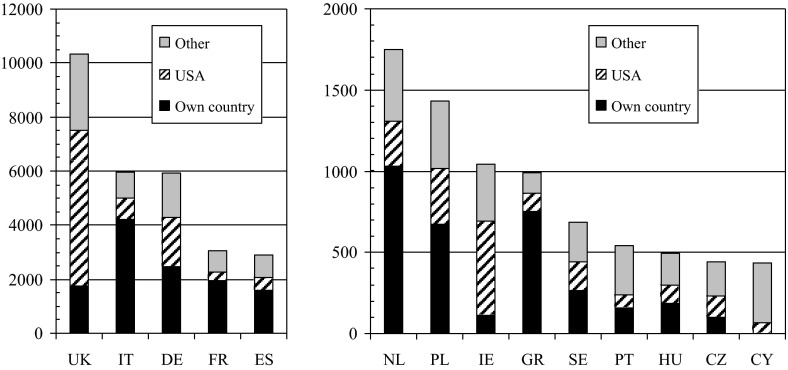
Locations of lung cancer researchers with names characteristic of different European countries—in own country, in the USA, and in other countries. For codes, see Table 1

We can also see where the lung cancer researchers with various “European” names are now—some will have stayed in their own country, some have gone to the US, and some have gone elsewhere. Figure 2 shows the situation. The five largest countries (in terms of numbers of named researchers) are on the left chart and the next nine are on the right chart. However, many of those with British, German, Polish and Irish names will be descendants of migrants resident in the USA often for several generations. The Greeks, followed by the Italians, French and the Dutch, are the most likely to remain in their own country. On the other hand, almost all Cypriot lung cancer researchers are now abroad, and most of the Czech, Portuguese and Hungarians have also left their own countries.

## Discussion

This paper greatly extends the methodology used in Roe et al. (2014) by its application to all the papers in a subject area, including multi-national ones, and by the provision of a file of all the named researchers, classified by their ethnicity and sex, and the country or countries in which they were working. This allows many research questions to be addressed, and some of them have been in this paper.

However the methodology still has some limitations, and these are currently being tackled. The first is that, although Aakre, J. can be identified as the same as Aakre, Jeremiah and so classed as male, the file often contains two separate entries (or three in this case because he also published a paper with a Chinese address) which should be amalgamated. The second limitation is that the number of each researcher’s papers is given only as an integer count, and for many purposes it would be more useful to have a fractional count, based on the number of different authors of each paper. This is sometimes problematic, as quite a lot of papers list individuals with more than one affiliation. This would not matter if these are all in the same country, as is usual, but increasingly nowadays senior researchers have appointments in more than one country. We would need to fractionate these people’s contributions by country in order to make the sum of the individual contributions equal the number of papers (less those with anonymous authors).

A further problem is that, although most names can be classed by country or region within it, at present some can not be. (The lung cancer database only has 392 names not classified by ethnicity, fewer than 0.5 % of the total.) This is well within the margin of error for most bibliometric studies. However, there is a bigger issue with ambiguous family names where the given names are not on the paper. We have approached this on the basis that most East Asians stay in their own country (see Table 7). It is not a fundamental problem, but it would require separate processing of the names in each individual country, a somewhat tedious procedure. However this method would not apply so well to Europeans, and as movement and marriage between EU Member States become increasingly common, there will be more errors in attribution of researchers to countries.

We have also found that the percentage of names that cannot be sexed is quite high, so that the results for some countries are not at all representative—notably for China. Clearly, we need to acquire more information on the sex associated with particular Chinese, Japanese and Korean names, although some names may not be strictly unisexual. (This occurs also with some European and some British given names, such as Hilary and Robin, where a minority of holders are respectively male and female.) We previously took a ratio of at least 10:1 as indicative of the association of a given name with just one sex, but there may be some errors, though these could be reduced if a researcher has two given names and one can be sexed definitively. This again will need improvements to the software.

The results for the distribution of authors by sex are by now almost wearily familiar, with a progressive attrition of women from the cadre of researchers at more senior (and productive) levels. Various solutions have been proposed, such as better provision of child-care facilities, more mentoring by successful female scientists, and more recently in our own institution some positive discrimination if individuals of apparently equal promise are being considered for a post. However it has to be recognised that other career options, such as scientific editing and publishing, may attract women because their demands are more predictable and so more compatible with family responsibilities. In the longer term, attitudes to women in research are changing, but it is perhaps unrealistic to expect major improvements to occur in a single generation. Methods such as the one described here will enable progress to be monitored and allow good practice in countries in the vanguard to be seen and then adopted more widely.

The situation with regard to ethnicity is inevitably bound up with immigration policy. It is clear from this and other studies that immigrants (here, people with names of foreign ethnicity or national origin) are making a substantial contribution to the scientific output of their host nations. Moreover, the active lobbying by universities and high-tech industry to relax barriers so that they can recruit from the best available talent is clear evidence of the value that they place on a diverse population of researchers. Indeed, the presence of well-qualified immigrants in a research lab is likely to generate novel ideas and approaches to difficult problems. The methods developed in this study could show whether an ethnically more diverse body of researchers in a country is correlated with higher impact research, and this is a study that we plan to initiate in the future.
